# Safety of Technique and Procedure of Stromal Vascular Fraction Therapy: From Liposuction to Cell Administration

**DOI:** 10.1155/2020/2863624

**Published:** 2020-07-04

**Authors:** Karina Karina, Iis Rosliana, Imam Rosadi, Rachel Schwartz, Siti Sobariah, Irsyah Afini, Tias Widyastuti, Melinda Remelia, Komang Ardi Wahyuningsih, Jeanne A. Pawitan

**Affiliations:** ^1^Klinik Hayandra, Yayasan Hayandra Peduli, Jl. Kramat VI No. 11, Jakarta Pusat, Indonesia; ^2^HayandraLab, Yayasan Hayandra Peduli, Jl. Kramat VI No. 11, Jakarta Pusat, Indonesia; ^3^Boston Children's Hospital, 300 Longwood Ave, Boston, MA, USA; ^4^Department of Basic Biomedicine, Faculty of Medicine, Universitas Kristen Indonesia, Jakarta, Indonesia; ^5^Department of Histology, Universitas Katolik Indonesia Atma Jaya, Jakarta, Indonesia; ^6^Department of Histology, Faculty of Medicine, Universitas Indonesia, Jl. Salemba 6, Jakarta Pusat, Indonesia; ^7^Stem Cell Medical Technology Integrated Service Unit, Dr. Cipto Mangunkusumo General Hospital/Faculty of Medicine, Universitas Indonesia, Jl. Diponegoro 71, Jakarta 10430, Indonesia; ^8^Stem Cell and Tissue Engineering Research Center, Indonesia Medical Education and Research Institute (IMERI), Faculty of Medicine, Universitas Indonesia, Jl. Salemba 6, Jakarta 10430, Indonesia

## Abstract

**Background:**

Stromal vascular fraction (SVF) therapy has been performed over the past six years to treat 421 patients by our group in five clinical centers. Autologous SVF, which is a substance containing stem cells, was isolated from lipoaspirate, mixed with platelet-rich plasma (PRP), and administered to patients with degenerative diseases, autoimmune diseases, trauma, aging, and other diseases with unknown etiology. This study aimed to determine the safety of SVF and PRP that were given through infusion, spinal, and intra-articular injection.

**Methods:**

The lipoaspirate was treated with a tissue-dissociating enzyme, and then, through centrifugation, SVF was isolated. In addition, blood was drawn from each patient, and PRP was isolated. Autologous PRP and SVF were administered to all subjects by intravenous (IV) injection. A minority group within the population received an additional spinal or intra-articular injection. The type of intervention was determined by each disease evaluation. The cell doses and adverse events for each patient were documented and analyzed.

**Results:**

Cell dose that was considered to be safe was less than 10 billion SVF cells in 250 cc of normal saline, for IV injection, and less than 1 billion SVF, for intra-articular and spinal injection. Adverse events were not severe and were treated successfully. Any observed adverse events were identified as a result of spinal or intra-articular injections and were not related to SVF or PRP.

**Conclusions:**

Our results showed that administration of high dose of SVF until 10 billion cells in a majority of 421 patients through infusion, spinal, and intra-articular injection was feasible without causing major adverse events and should be further investigated in well-designed phase I-II clinical trial to address the safety and efficacy of therapy.

## 1. Introduction

Mesenchymal stem cells (MSCs) can be isolated from adipose tissue from patients through a simple liposuction technique [1, 2]. MSCs are particularly useful for regenerative healing because they are multipotent cells, which can differentiate into a variety of cell types [3]. After lipoaspirate processing, MSCs are obtained along with endothelial cells, macrophages, pericytes, and T cells, which together comprise a substance referred to as stromal vascular fraction (SVF), in a relative short time, usually in 2-3 hours [4, 5]. Culturing SVF leads to MSC isolation, and 1-2 week-culture period is needed to produce millions of these fibroblast-like plastic adherent cells known as MSCs [6]. Autologous SVF extraction procedure replaces any need for allogeneic cell culturing [7], and therefore risks of contamination, aging of stem cells, or rejection can be avoided [8–10]. Moreover, the procedure is less expensive. Therefore, many therapies were performed using autologous SVF rather than autologous or allogeneic MSCs [7].

Recently, researchers reported immunomodulation effect of MSCs in experimental autoimmune disease. MSCs can inhibit proliferation of T cell from lymph nodes of encephalomyelitis, reduce IFN-*γ* production by T cell, and significantly inhibit total antigen specific IgG production, as well as that of each IgG subclass [11]. MSCs also secrete various factors to reduce proinflammatory cytokine such IL-6 and TNF*α* that may be useful for treating inflammatory diseases such as cystic fibrosis lung disease [12]. It is well known that MSCs secrete proangiogenic cytokines, such as vascular endothelial growth factor (VEGF), angiopoietin-1 (Angpt-1), hepatocyte growth factor (HGF), and insulin-like growth factor-binding proteins (IGFBPs) to promote cells regeneration [13]. To date, stromal or mesenchymal stem cell application has been used to treat many diseases and conditions, including osteoarthritis [14], rheumatoid arthritis [15], chronic obstructive pulmonary disease (COPD) [16], heart disease, Lyme disease, Crohn's disease/colitis, autoimmune diseases, diabetes, lower limb vascular disease, kidney failure, neurological symptoms, degenerative disc disease, vaginal dryness, urinary incontinence, hair loss and alopecia [17], fat grafting [18], multiple sclerosis [19], and retinitis pigmentosa [20] and for aesthetic purposes [21].

Platelet-rich plasma (PRP) has high growth factors content [6, 7]. In *in vitro* studies, PRP was used as the source of growth factors to support MSC proliferation and differentiation [6, 22–25], replacing the use of animal-derived serum. In animal and human studies, PRP was used for accelerating wound healing process [26], improving regeneration of injured articular cartilage [6, 7], revising hypertrophic scars [21], and reducing the necrosis that frequently occurred in fat graft procedure [27].

Most researchers showed safety and potential benefits of SVF therapy, and all collected data indicated evidence of a safe procedure. In fact, SVF and PRP therapy have been performed in Indonesia since 2011. However, Indonesian recent regulation in 2018 required any cell therapy, including SVF therapy, to be conducted on the basis of clinical trial in order to prove safety and efficacy. In this article, we report our experience in combining SVF and PRP therapy since 2011 to treat degenerative diseases, autoimmune diseases, trauma, and other diseases, in addition to using them as antiaging therapy. Those SVF and PRP were processed using several methods that include the use of commercial kit and laboratory-based developed methods. The purpose of this article is to provide supportive knowledge regarding the process of SVF and PRP preparation for clinical application; the highest dose of SVF has been administered through infusion, spinal, and intra-articular injection without tolerable side effects. In addition, we present precautions for avoiding major unwanted side effects in future clinical trials investigating the safety and efficacy of this therapy in certain diseases.

## 2. Methods

This was a retrospective study involving patients that were treated with autologous SVF and PRP for a variety of conditions. The fat aspiration procedures were mostly performed by a single operator in five clinical sites in Jakarta, Indonesia. Most of the administration procedures were done in Hayandra Clinic by the physician with specific competencies (internist, neurologist, plastic surgeon, and antiaging doctors). Ethical clearance was obtained from Health Research Ethics Committee, University of Indonesia, and Cipto Mangunkusumo Hospital (HREC-FMUI/CMH) with letter of approval No. 0249/UN2.F1/ETIK/2018. Previously recorded medical data was collected from the medical records documented by the clinics or hospitals.

### 2.1. Group of Pathology

Patients were divided into 5 groups, namely, involving antiaging, degenerative diseases, autoimmune diseases, trauma, and unknown etiology such as hearing loss. Degenerative diseases involved diabetes mellitus, degenerative musculoskeletal disease, cardiovascular and metabolic disease, stroke, brain disorder, respiratory disease, renal failure, degenerative brain disease, eye disease, neurological disorder, and reproductive system, while autoimmune diseases involved autism and autoimmune disease.

### 2.2. SVF Processing Method

SVF is a heterogeneous mixture of cells that includes endothelial cells, erythrocytes, fibroblasts, macrophages or other immune cells, progenitor, pericytes, and also MSCs [5]. In this study, SVF refers to all cell types in the fraction. SVF was processed using three different methods, referred to as Methods 1, 2, and 3. Adipose tissue was collected from abdominal fat tissue for adults and thigh paediatric for children (aged below 15 years). Liposuction technique was aspirated manually using 2.4 mm cannula with tumescent technique for anesthesia.

In Method 1, SVF was isolated from lipoaspirate by commercial kit as described by the manufacturer. Briefly, about 15–600 mL of fat was collected by syringe and stored in 50 mL tube. SVF cells were extracted from fat using the extraction kits according to the manufacturer's guideline. Fats were washed 3 times with saline solution and then incubated with commercial enzyme for 30 minutes. The sample was centrifuged for 10 min, 3000 rpm, to collect SVF as pellet at the bottom of the tube. The cells were filtered using 100 *µ*m pore size cell strainer (Gibco, USA). To collect the SVF, cells were then centrifuged for 10 min, 3000 rpm, at room temperature.

Method 2 is available method provided by a stem cell laboratory in Jakarta. About 15–600 mL of fat was stored in 50 mL tube and then washed with saline solution. The fat tissues were then digested by in house enzyme for 1 hour at 37°C. The fats were washed with saline solution, followed by centrifugation for 5 min at 600 ×g. The supernatant was discarded to get SVF.

Method 3 was invented by HayandraLab, as an improvement to the previous method, and was referred to as the H-Remedy method (patent application no. P00201603083) [28]. Lipoaspirates (about 15–600 mL) were digested by H-Remedy enzyme and incubated for 1 hour at 37°C, 300 rpm. After incubation, the digested lipoaspirates were added to low-glucose (1 g/L) Dulbecco's modified Eagle's medium (DMEM) containing 4 mM L glutamine (Gibco, USA) to inactivate the enzyme, followed by centrifugation for 5 min at 600 ×g. Then, the supernatant was discarded. The SVF pellet was diluted in saline solution. The cell number and viability were counted by trypan blue staining. Calculation of live cells used the following formula:

(1) $$\begin{array}{c} \text{Number of cells}=\frac{\text{average live or dead cells}}{4\left(\text{chambers}\right)}\times \text{Df}\times 10^{4}\times \text{cell suspension volume,} \end{array}$$

where Df is the dilution factor.

The following formula was used to calculate cell viability:

(2) $$\begin{array}{c} \text{Cell viability}=\frac{\text{average live cells}}{\text{average live}+\text{dead cells}}\times 100\%. \end{array}$$

In our previous publications, SVF isolated using each method has been characterized. Cultured SVF is reported to express CD73, CD90, and CD105 without or with less expression of CD34/CD45/CD11b/CD19/HLA-DR, which are able to differentiate into chondrocyte, osteocyte, and adipocyte that meet criteria of MSCs as defined by the International Society of Cellular Therapy [24, 25].

### 2.3. Platelet-Rich Plasma Processing Method

Platelet-rich plasma (PRP) was prepared using two different methods: the commercial kit (Platelet-Rich Plasma Kit, AdiStem Ltd., Hong Kong) and the conventional method, which was invented and developed by HayandraLab. In brief, 24 cc samples of whole blood were collected from each respective patient, in a sodium citrate tube, and centrifuged at low speed for 5 minutes until the plasma layer separated from the red blood cell (RBC) layer. Plasma was aspirated, collected, and subjected to second centrifugation step, requiring high speed for another 5 minutes until platelets were concentrated at the bottom of the tube. Plasma was removed until the final volume of plasma remaining in the tube was 3 cc. The pellet of platelets was resuspended in the remaining plasma in the tube (3 cc), which was regarded as inactivated PRP. This 3 cc of PRP was then activated using calcium chloride solution until a clot was formed. Clots were removed to isolate the PRP, and the PRP was subjected to light activation (AdiLight-1, AdiStem Ltd., Hong Kong). The laser-activated PRP was used in combination with SVF. The light activation was conducted to increase anti-inflammatory cytokine, such as interleukin-1 receptor antagonist (IL1-RA) levels in PRP [29]. This protocol was applied in Methods 1, 2, and 3.

It was noted, during the use of commercial kit, that the protocol of PRP preparation was changed based on the instruction from the kit manufacturer. The newest protocol of Method 1 demonstrated that there was no need for PRP activation using calcium chloride. However, our group found that clot formation and removal from PRP were important in the case of intravenous injection, which demanded a different protocol to generate the most safe and effective therapy.

### 2.4. SVF Administration

The SVF pellet was resuspended in 0.9% normal saline, resulting in a total of 22 mL SVF suspension. We used 7 mL of cell suspension for quality control (contamination, endotoxin, and MSCs culture). A total of 15 mL SVF suspension was mixed with 3 mL autologous PRP that had been activated previously using calcium chloride solution, and light therapy by AdiStem Ltd. Photobiostimulation. A total of 20 mL SVF and PRP suspension was injected into an infusion bag, which contained 250 mL of 0.9% normal saline, if the total cell number was more than 1 billion. If the cell number was less than 1 billion, the normal saline volume was 100 mL. The mixture of SVF and PRP together was then infused intravenously to each patient over duration of 30 minutes. Patients were treated with a range between 1 and 3 separate SVF-PRP infusions. Time at which patients received SVF-PRP procedures also determines numbers of infusions since sufficient number of SVFs for repeated infusions was only received after SVF processing was performed using H-Remedy method. In addition, three repeated infusions were only done after we validated a method to store SVF in nitrogen liquid tank and to thaw the cells while still resulting in sufficient number of cells that is more than 100 million cells for the third infusion. Number of repeated injections also depends on the case and needs of each patient, as determined by patient medical conditions, such as severity of illness and disease duration. For example, patients with disease duration more than a year or with moderate to severe diseases were given at least two infusions. However, if cell number is sufficient for three infusions, patients not meeting those criteria also received three infusions. The first infusion was done on the day of their liposuction procedure, followed by another infusion in two weeks apart.

Patients with osteoarthritis received additional intra-articular injections, with a total of 3 mL in each affected knee, which made the treatment more closely focused on a specific area. Patients who had presented with cerebral palsy, autism, stroke, Parkinson's disease, and dementia also received one spinal injection of SVF on the day of their liposuction procedure, at the same time of receiving their first infusion of SVF intravenously, in order to augment the strength and force of the treatment. In the cases which received an additional injection, 3 mL of the SVF and PRP mixture collected was used for spinal injection for autism and brain-related disorder or intra-articular injection for osteoarthritis patients. The remaining cell suspension was injected into the infusion bag containing 250 mL of 0.9% normal saline, or 100 mL depending on the SVF number.

### 2.5. Data Collection and Analysis

A retrospective analysis was performed on 421 SVF-PRP-treated patients, a patient population collected from September 2011 to August 2018. Variables included the specific conditions (gender, age, pathological conditions), SVF isolation method, repetition of SVF treatment, SVF dose, method of infusion, location of infusion, other treatments performed, and whether there was any incidence of adverse reactions. Side effects or patient discomforts or complaints were recorded as adverse events (AEs). According to similar study conducted by Comella et al. [18], any event that is life-threatening and led to hospitalization or required major medical intervention is categorized as serious adverse events (SAEs). All other events, side effects, or patient complaints/discomforts were collected as adverse events (AEs). Data was grouped according to pathological condition and further analyzed descriptively, as seen in the figure and tables included.

## 3. Results

### 3.1. Patient Demographics and SVF Procedure

Among 421 patients, 194 patients (46%) were male and 227 (54%) patients were female. The median age was 55 years old. The youngest patient studied was 1 year old, and the oldest patient was 86 years old.

**Figure 1:**
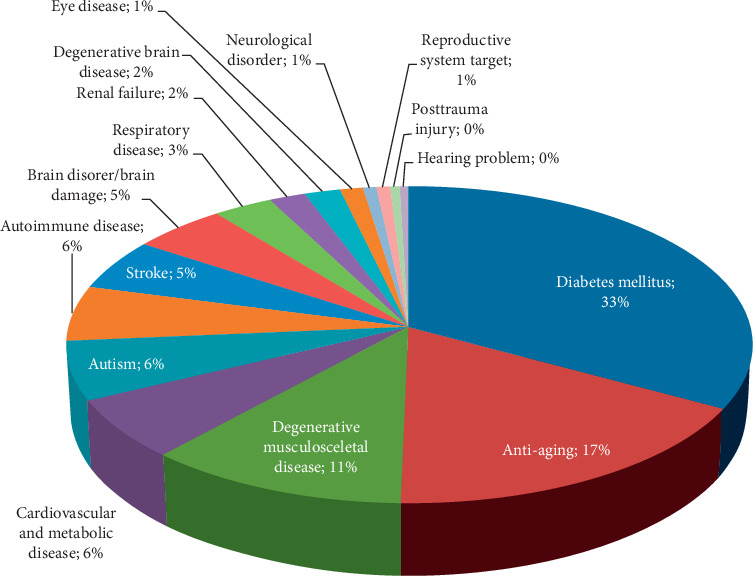
The pathological patients' conditions: there were sixteen groups which were diabetes mellitus (140), antiaging (72), degenerative musculoskeletal disease (47), cardiovascular and metabolic disease (26), autism (25), autoimmune disease (24), stroke (22), brain disorder (21), respiratory disease (13), renal failure (8), degenerative brain disease (8), eye disease (5), neurological disorder (3), reproductive system (3), trauma posttrauma injury (2), and hearing problems (2).

Patients were divided into sixteen groups based on their pathological conditions (Figure 1). The full distribution of categories, including the number of patients, age, gender, volume of fat cells processed, SVF processing method used, number of repetitions of treatment, cell dose given, and route of administration per disease group, is presented in Table 1.

Disease groups were sorted from the most to the least common cases, as per percentage values. The top 5 common diseases during the study were diabetes mellitus, antiaging, degenerative musculoskeletal disease, cardiovascular and metabolic disease, and autism. Other treated diseases can be seen in Table 1.

SVF was mostly isolated using H-Remedy method (330 patients, 78%), followed by commercial kit (82 patients, 19%) and then available method (9 patients, 3%) being the least used method. All patients received SVF through IV administration, while some received additional intra-articular and spinal injection, depending on the disease and condition, which has been taken into consideration during the overall evaluation. All patients who were treated during the period when Method 1 and Method 2 were being used received only one treatment. Method 3 implemented additional series of infusions, which was important in ultimate healing and treatment progress, which was observed in the success of the treatments, as documented. From a group of 330 patients, 193 patients received three series of infusion, 123 patients received one infusion only, and 105 patients received two infusions. Each infusion was received in 2 weeks apart. After cell counting, the viability of isolated SVF from all patients was more than 90%, regardless of SVF isolation method used, which was important for the efficacy and safety of the treatment. The distribution of cell doses administered via IV, intra-articular, and spinal injection is presented in Table 2.

### 3.2. Intravenous Injection

The concentrations of SVFs within the first infusion received by 421 patients varied between 1 million to 26.12 billion cells. A large portion of patients received between 1 and 5 billion SVFs (221 patients, 52%), followed by the next majority group who received 100 million to 1 billion SVFs (73 patients, 17%) and the last majority group having received 5−10 billion SVFs (53 patients, 13%). Only 23 patients (5%) received less than 100 million SVFs. Seven patients (2%) received more than 10 billion SVFs in first infusion. SVF records were not available for 44 patients (11%) in Method 1 group, which was one of the limitations for this study. The number of cells infused was dependent on the isolation process and the unique composition of SVFs within adipose tissue of each individual. Overall, the median dose of cell population within the first SVF infusion was calculated as 1.93 billion.

For the second SVF infusion, which was received by 298 patients, the number of injected SVFs varied between 0.03 billion and 9.64 billion cells. Half of the patients received 1−5 billion SVFs (147 patients, 50%), followed by 100 million to 1 billion SVFs (138 patients, 46%). Nine patients (3%) received SVFs ranging from 5 to 10 billion cells, and 4 patients (1%) received fewer than 100 million SVFs. The median number of SVFs injected was 1.02 billion cells.

In the cases of patients who received three SVF infusions (193 patients), the number of SVFs injected during the third and final treatment ranged between 0.10 billion and 3.04 billion cells. Seventy-four percent of patients (143 patients) received between 100 million and 1 billion SVFs, and the remaining patients (50 patients) received between 1 and 5 billion SVFs. No patients received an SVF infusion containing less than 100 million cells or more than 5 billion cells. Overall, the median number of cells within the SVF at the 3^rd^ infusion was 0.64 billion cells. The number of cells infused has been compared to the efficacy and overall outcome of each patient's treatment progression.

### 3.3. Intra-Articular Injection

A total of 47 patients who had presented with osteoarthritis and other musculoskeletal diseases, specifically concerning the knees, received intra-articular injections of SVF in the affected areas. The number of SVFs injected into the affected knees varied from 1 million to 1.60 billion cells. Patients commonly received less than 1 billion cells (27 patients, 57%). The median dose of SVFs for intra-articular injections was 0.17 billion SVFs per each affected knee. Thirteen patients (28%) received less than 100 million cells, and only 3 patients (6%) received 1 to 5 billion cells. Data was lost for 4 osteoarthritis patients (9%).

### 3.4. Spinal Injection

In total, seventy-six patients received SVF through spinal injections. The median number of cells administered was 0.28 billion, ranging from 0.001 billion cells to 4.12 billion cells. More than half of the patients (47 patients, 62%) received less than 1 billion SVFs, followed by patients who received less than 100 million SVFs (13 patients, 17%). Only three patients (4%) received between 1 and 5 billion SVFs, which was the smallest amount administered. Data was lost for 13 patients (17%) during the use of Method 1.

### 3.5. Safety Analysis and Management

All of evaluated patients experienced immediate hematoma growth surrounding the entry site for liposuction procedure and general inflammation. Mild analgesics, taken three times daily for a three-day duration, were used to provide relief of symptoms reported, including hematoma growth, inflammation, and mild to moderate pain and discomfort. Out of all patients recorded, two patients reported uniquely extreme discomfort at the liposuction site, which was not relieved within the initial 3 days. These patients in particular were advised to continue the analgesic regimen for an additional two weeks. For the two patients who required additional pain management, all symptoms were relieved within the following two weeks of treatment, using mild pain killers.

11 patients (3%) reported that they experienced shivering, which lasted for several minutes after procedure and diminished after 1 ampoule of corticosteroid was administered through IV infusion. This side effect was only reported during the period when SVF was isolated using Method 1 techniques, which contributed towards the termination of this method being used. Patients were prescribed a 3-day oral medication regimen, immediately following surgery, including either paracetamol or ketorolac and cefixime or ciprofloxacin, as was decided for each individual patient. The SVF from those 11 patients was processed using the same batch of kits provided by Method 1 processing manufacturers. Microbial tests were performed, to check for sterility of the kits, by an authorized laboratory. These tests confirmed that the reagent (used to dissociate the fat tissue and free the stem cells) from the suspected batch, which produced adverse results, was contaminated by *Enterobacter cloacae* and *Klebsiella oxytoca* bacterium. The manufacturer had released nonsterile processing kits due to mal-production, and therefore our group switched from using Method 1 to using a new method, referred to as Method 2.

During the time when Method 1 was used, one patient experienced thrombus formation in left anterior inferior cerebellar artery. This patient received nonactivated PRP administered by IV injection, due to the change of the protocol of the manufacturer of the commercial PRP kit. This patient recovered ultimately after successful thrombus removal treatment, which was performed in the hospital. After this incident, all of the patients received PRP that has been activated by calcium chloride solution, which gave successful outcomes with all of them. Among the patient population (76 patients) who have been treated with a spinal injection, five patients (7%) reported to have experienced headaches and discomfort, which lasted within the first week following the injection. All of the patients found relief of symptoms within one week of beginning analgesic treatment.

## 4. Discussion

Considering available publications that were reviewed, it is believed that our group is the first to treat patients with the largest dose of SVF concentrations recorded, administered through more than one route, including intravenous (IV), intra-articular (IA), and spinal injection. In practice, we used calcium chloride–light irradiation–activated PRP that is safe for IV administration, which augmented the therapy and produced better results for the patients. Previous studies had proved that light activation reduces production of proinflammatory interleukin-1a, IL-2, IL-5, and IL-6 and increases anti-inflammatory factor, IL-10, secretion in T cell culture [30]. The benefit of using light irradiation to activate PRP was reported in osteoarthritis patients [31]. Rodriguez reported the treatment of 12 rheumatoid arthritis patients using SVF containing 16−45 million cells per IV injection, and each patient received a maximum of four injections. However, there was no clear explanation for the volume of saline solution or the time of infusion in this study [15]. In another phase I clinical trial, conducted by Comella et al., twelve patients with end-stage COPD were treated with one single SVF infusion [32]. The range of SVF dosing given to each patient varied from 150 to 300 million cells, in combination with 1000 mL saline solution. Infusion was administered over the course of 2 hours, via the antecubital vein. Another multicenter study was published by the same author group, Comella et al., in which 246 patients were treated with autologous SVF and PRP [18]. The systemic symptoms and diseases that were treated included COPD, heart disease, Lyme disease, Crohn's disease/colitis, various autoimmune diseases, diabetes, and kidney failure [18]. SVF was administered by IV injection throughout a duration of 30−60 minutes via bolus push, and the cell dose was between 30 and 60 million cells in 3−5 mL of 0.9% normal saline. Another 3−5 mL of normal saline was infused to clear the line. In the same study, 93 patients who had presented with neurological symptoms were treated with SVF intrathecally via lumbar puncture, 33 patients with degenerative disc disease were treated with 1 mL of SVF and PRP mixture by an intradiscal injection, 264 patients with orthopedic symptoms received intra-articular SVF and PRP injection (3−5 mL), 7 patients received SVF and PRP injection into the scalp for hair loss, and 1 patient received intramuscular SVF and PRP injection of 5−10 mL mixture of SVF and PRP into their legs to target vascular disease. Several other patients received SVF infusion, along with a fat transfer procedure, and a total of five patients received injections of 5 mL SVF and PRP in their sex organs for degenerative conditions [18]. Riordan et al. published three case reports of SVF therapy for multiple sclerosis patients [33]. Three patients received two IV injections of 25, 28, and 75 million SVFs every 10 days, followed by multiple intrathecal and IV infusions of allogeneic CD34+ and MSCs [33].

We observed that a majority of these published studies used not more than 100 million SVFs, except one study which was done by Comella et al. [32]. It is hypothesized that the fundamental reason for the use of this smaller amount of injected SVFs in clinical application is likely the inability of investigators to isolate large number of cells from adipose tissue collected from their patients. Most of the stem cell studies also used this general number range of stem cells, likely due to high costs and cumbersome technique necessary to provide large number of stem cells [34]. One exception was particular autologous human adipose-derived MSCs (ADMSCs) study by Ra et al. in spinal cord injury (SCI) patients [35]. In this ADMSCs study, eight SCI patients were treated with one IV infusion of 400 million autologous ADMSCs. The cells were divided into four doses, resulting in one infusion bag which consisted of 100 million cells per 100 mL normal saline (1 million cells/mL). The cells were then injected into the cephalic vein over the course of 3 to 4 h [35].

We considered that determining the optimal dose is a crucial step for establishing an effective cell therapy. It is generally expected that number of cells administered will be proportionate to the efficacy. Koga et al. reported that higher number of implanted rabbit synovium-derived MSCs improved cartilage regeneration in animal model [36]. Another previous study reported that the amounts of cartilage matrix and proteoglycan were higher in composites consisting of 5 × 10^7^ cells than those consisting of lower cell densities (1 × 10^6^ and 1 × 10^7^ MSCs) [37]. However, these findings were not consistent with other studies. In a dose escalation study reported by Park et al., efficacy of human umbilical cord blood-derived MSCs for cartilage repair was higher in lower MSC doses (0.1, 0.5, and 1 × 10^7^ cells) than those in higher dose (1.5 × 10^7^ cells) [38]. It might be proper to consider several factors such as the source of the cells, their characteristic, and route and delivery vehicle for cell administration, as well as patient condition, in determining the optimal dose in stem cell therapy. Based on our clinical experience, greater improvements for patient symptomatology were achieved when patients received higher number of SVFs. It is plausible that when intravenously administered, the stem cells traveling through the bloodstream are distributed to a less concentrated area of tissues, resulting in only low detectable level of injected cells in target tissue, compared with that of intra-articular injected cells, in one specific region of the body [39]. Moreover, we have confirmed in our previous *in vitro* research that culture SVF isolated from diabetic patients formed less colonies than those isolated from nondiabetic patients [40]. Similar results were obtained from MSCs from aged donors that also show reduced viability, cell growth, increase of cell senescence, and superoxide dismutase (SOD) activity [41]. Therefore, it is important that the number of viable SVFs is as high as possible, so that the injected cells can reach target tissue in an optimum number to compensate for the reduced biological function of cells from aging or degenerative diseases, thus being most effective for regeneration.

The next thing to consider in determining the dose in cell therapy is the safety of the cell dose for the patient. In this study, in all cases, the majority of patients received less than 10 billion SVFs. We noted that 6 out of 7 patients who received more than 10 billion SVFs did not report any incidence of discomfort or any side effects. However, we consider that the highest number of SVFs which can be infused to the patient is 20 billion cells, to avoid the cytokine storm-like side effect.

In the case of spinal injection, 8% of the patients experienced discomfort and incidence of experiencing headaches following spinal injection. Headaches may have been experienced following spinal injections due to repetitive insertion and removal of the spinal needle. Upon repetitive puncturing of the spinal cord, a leak in the dura becomes more likely. In the case of a leak in the dura, if any small amount of cerebrospinal fluid escapes, cushioning around the brain is reduced, and therefore a lower pressure system surrounding the brain causes moderate headaches. This symptom would continue while the dura heals. Patients who reported discomfort and headaches were prescribed mild analgesic medication and found relief of symptoms within one week of beginning mild treatment. Given uncomplicated symptom resolution, the evaluated symptoms can be associated with incidence of imprecise needle insertion instead of the cell doses, since the majority of cell doses for spinal injection are less than 1 billion. Imprecise needle insertion can be considered a human error, which would not concern the treatment itself.

Based on our previous study, we also notice that protocol for isolating the cells should be feasible, optimum for cells isolation (number and viability) [42], and also free of contamination. The reported median viability of SVF was higher than the minimum acceptable viability specification for somatic cell therapy, generally set at 70% [43]. Viability of cell product more than 70% has not demonstrated any adverse effects on the safety and efficacy of product administration for therapy. In this study, all SVFs processed have viability more than 90%, so there are no negative side effects reported as outcome for low viability cells in this study.

The most prevalent incidence of reported side effects is experiencing shivering, which was determined as being due to contamination from the kit of Method 1. It became important to test the sterility of the kit or reagent used for cell isolation, as it became clear that the manufacturer could not guarantee kit sterility. It was determined that one advantage of using Method 3 isolation protocol, developed at HayandraLab, is that we ensure that each material and treatment method is at minimum risk of contamination, and it is manageable to ensure sterility and safety.

We are aware that one source of contamination throughout the procedure could be the lipoaspirate collection itself, due to the presence of skin bacteria. In taking steps for reducing and preventing any serious side effects, related to bacteria contamination, we injected each patient with preoperative antibiotics (third-generation cephalosporins) and prescribed each patient a 3-day oral medication regimen immediately following the procedure, consisting of NSAID and antibiotic medications. Another opportunity for contamination to be aware of is tumor contamination. Garcia et al. found minimal tumor contamination present in their human MSC culture [44]. In our practice, SVF was combined with PRP, which is richly dense with various growth factors. Tumor cells can utilize these growth factors to support their proliferation and migration. Considering that there is no conclusive result present today about bimodal effect of stem cells on cancer patient, we considered that noncancer patient selection is important in the case of autologous SVF and PRP therapy, in order to find the most appropriate candidates for this treatment, to ensure safety, and identify patients who will benefit the most.

Our study showed that SVF therapy is a feasible, effective, and safe treatment method. As an autologous SVF therapy, SVF therapy proposes no specific threats, as demonstrated from 421 accounts of patient records. The cell dose that is considered to be tolerable with no patient's complaint or discomfort was a concentration of less than 10 billion SVFs in 250 cc of normal saline, for IV injection, and less than 1 billion SVFs, for intra-articular and spinal injection. We recommend that volume of saline solution used for IV injection and rate of infusion should be proportionate to the SVF number. A higher number of SVFs, over 20 billion cells, was considered to be potentially overwhelming and harmful to the patient and has not been tested. The majority of reported adverse events in this study were low risk, manageable, temporary, and expected outcomes of the SVF procedure. SVF can be combined with PRP in clinical practice and is beneficial to the outcome of the patients' recovery, as found through clinical implementation.

PRP contains highly concentrated platelets. Marx reported that double centrifugation of autologous blood can produce PRP with concentration of platelets of at least 1 × 10^6^ cell/*µ*L that is almost 5 times higher than the platelet concentration in the whole blood [45]. Platelets from majority of our patients, especially those who have diabetes, were easier to aggregate after second centrifugation as we observed in laboratory, which may reflect their behavior in blood circulation [46]. Aggregated platelet releases various cytokines and growth factors that in turn activate endothelium [47]. Activated endothelium recruits more platelets and circulating immune cells to adhere to and form plaque. Plaques that are ruptured can damage endothelium causing thrombus formation and vascular occlusion that might happen in one of our patients who received PRP without prior ex vivo platelet activation. Thus, for IV injection, PRP should be activated prior to infusion in order to avoid thrombus formation. This order of injection has demonstrated evidence of success and has not caused thrombus formation. In SVF-PRP therapy, noncancer patient screening is needed to avoid tumor contamination within SVF and has been effective in the present clinical treatment.

Our results showed that administration of high dose of SVF until 10 billion cells in a majority of 421 patients through infusion, spinal, and intra-articular injection was feasible without causing major adverse events. However, our study was done in retrospective manner, was not designed as a dose escalating study, and lacks some clinical data to draw reliable conclusion for determining the highest dose of SVF that is safe for clinical application. While phase I-II clinical study is sorely needed to address the highest tolerable dose of SVF, repetition, and safety, as well as potential benefit of this therapy in certain diseases, our study still offers some knowledge for researchers and clinicians working in the same area to improve their procedures, such as SVF-PRP processing and therapy.

## 5. Conclusions

Our results showed that administration of high dose of SVF until 10 billion cells in a majority of 421 patients through infusion, spinal, and intra-articular injection was feasible without causing major adverse events and should be further investigated in well-designed phase I-II clinical trial to address the safety and efficacy of the therapy.

## Figures and Tables

**Table 1:** Distribution of patients' conditions (age, gender), median of fat tissue processed, SVF processing method, repetition of treatment, and cell dose for IV injection based on pathological condition.

Group of disease	Pathological condition	Number of patients				Median of age, min and max (year)	Median of fat tissue volume, min and max (cc)	Number of patients						Median of total cell dose, min and max (billion) for IV	
								SVF processing method			Repetition of treatment				
		*N*	%	M	F			1	2	3	1	2	3	1	2	3
Diabetes mellitus	DM type 1 and 2	140	33.25	93	47	56 (13–85)	145 (20–393)	20	4	116	33	38	69	2.42 (0.003–19.24)	1.00 (0.03–8.48)	0.72 (0.13–2.92)
Antiaging	Systemic rejuvenation	72	17.10	19	53	51 (34–84)	130 (15–613)	9	1	62	22	18	32	2.08 (0.20–11.80)	1.02 (0.10–8.12)	0.62 (0.18–2.08)
Degenerative musculoskeletal disease	Osteoarthritis, osteoporosis, and herniated nucleus pulposus	47	11.16	14	33	64 (10–86)	183 (50–445)	11	1	35	13	11	23	1.11 (0.001–10.12)	0.92 (0.15–9.64)	0.44 (0.10–2.40)
Cardiovascular and metabolic disease	Cardiomyopathy, hypertension, postcardiac stenting	26	6.18	19	7	57 (4–71)	181 (45–445)	7	0	19	8	10	8	2.00 (0.04–8.18)	0.97 (0.11–5.07)	0.88 (0.21–2.10)
Autism	Autism	25	5.94	18	7	10 (5–34)	108 (30–326)	11	0	14	12	5	8	2.04 (0.001–4.24)	1.57 (0.33–3.92)	0.95 (0.13–1.64)
Autoimmune disease	Multiple sclerosis, myasthenia gravis, psoriasis, and vasculitis	24	5.70	6	18	41 (16–69)	101 (30–323)	6	0	18	6	5	13	2.30 (0.02–7.25)	1.12 (0.08–2.96)	0.69 (0.32–3.04)
Stroke	Chronic ischemic stroke	22	5.23	14	8	57 (32–75)	120 (40–399)	2	0	20	4	5	13	1.47 (0.40–19.60)	0.82 (0.08–2.64)	0.63 (0.10–2.65)
Brain disorder/brain damage	Epilepsy, cerebral palsy, postmeningitis related brain injury, posttraumatic brain damage, post-choriocarcinoma	21	4.99	16	5	12 (1–44)	65 (15–375)	4	0	17	7	7	7	1.52 (0.04–26.12)	1.20 (0.20–3.82)	0.59 (0.14–1.08)
Respiratory disease	COPD	13	3.09	10	3	68 (48–78)	230 (58–349)	7	0	6	7	2	4	1.39 (0.002–8.82)	1.77 (0.60–5.84)	0.61 (0.40–1.28)
Renal failure	Chronic renal failure	8	1.90	5	3	67 (45–76)	209 (93–305)	3	0	5	3	1	4	1.74 (1.16–4.17)	1.73 (1.20–3.94)	0.76 (0.51–1.39)
Degenerative brain disease	Parkinson's, dementia, Alzheimer	8	1.90	6	2	61 (55–77)	98 (60–200)	0	3	5	3	0	5	1.22 (0.24–2.68)	0.28 (0.22–0.83)	0.49 (0.13–1.03)
Eye disease	Macular degeneration, retinitis pigmentosa	5	1.19	3	2	71 (49–77)	120 (80–200)	2	0	3	2	1	2	1.04 (0.14–3.05)	0.44 (0.33–0.82)	0.66 (0.31–1.01)
Neurological disorder	Mental retardation, hypoconcentration	3	0.71	1	2	27 (21–55)	60 (37–120)	0	0	3	2	0	1	1.96 (0.31–2.24)	0.16	0.10
Reproductive system target	Improving quality of sperm, climacteric	3	0.71	1	2	62 (35–62)	120 (100–285)	0	0	3	0	1	2	1.64 (1.43–2.13)	1.32 (0.45–2.76)	0.63 (0.55–0.71)
Posttrauma injury	Multiple fracture, urethrae rupture, and skin loss after trauma	2	0.48	1	1	43 (17–69)	121 (120–121)	0	0	2	1	0	1	4.81 (1.44–8.18)	0.44	0.48
Hearing problem	Deafness and hearing loss	2	0.48	1	1	36 (22–50)	179 (130–228)	0	0	2	0	1	1	3.80 (2.31–5.29)	1.90 (1.16–2.64)	0.77
Total		421	100	194	227			82	9	330	123	105	193			

Method 1: SVF was isolated from lipoaspirate by commercial kit. Method 2: available method provided by stem cell laboratory in Jakarta. Method 3: H-Remedy established by HayandraLab.

**Table 2:** Cell doses for IV, intra-articular, and spinal injection.

	Median (min–max) cells dosage (billion)	Number of patients						
		<100 million cells	<1 billion cells	1 to 5 billion cells	5 to 10 billion cells	>10 billion cells	No data	Total
1^st^ infusion	1.93 (0.001–26.12)	23	73	221	53	7	44	421
2^nd^ infusion	1.02 (0.03–9.64)	4	138	147	9	0	0	298
3^rd^ infusion	0.64 (0.10–3.04)	0	143	50	0	0	0	193
Intra-articular injection	0.17 (0.001–1.60)	13	27	3	0	0	4	47
Spinal injection	0.28 (0.001–4.12)	13	47	3	0	0	13	76

Patients with osteoarthritis and other degenerative musculoskeletal diseases in knees got additional intra-articular injection, and those who had presented with cerebral palsy, autism, stroke, Parkinson's disease, and dementia also got additional spinal injection of SVF on the day of first infusion.

## Data Availability

The data used to support the findings of this study are included within the article.

## Abbreviations

ADMSCs: Adipose-derived MSCs
COPD: Chronic obstructive pulmonary disease
IA: Intra-articular
IV: Intravenous
MSCs: Mesenchymal stem cells
PRP: Platelet-rich plasma
SCI: Spinal cord injury
SVF: Stromal vascular fraction.
